# Typhoid Fever, with Threatened Coxalgia Supervening on Recovery

**Published:** 1861-07

**Authors:** 


II. *Typhoid Fever, with threatened Coxalgia supervening on Recovery.*—J. D., an English lad, aged nineteen, was admitted May 16th. Attention was again called to his case, which had previously occupied a portion of a clinique in a consideration of the disease—typhoid fever—under which he was then laboring; but now, during his convalescence, a new and interesting feature had presented itself.

The history of the case, as given at the bedside, is briefly as follows: For a week or ten days, previous to his admission, he had been feeling unwell, with the ordinary, well-known symptoms—loss of appetite, some headache and pain of a dull, heavy character in the back and limbs, occasional nausea, with slight creeping chills and a general feeling of lassitude and weariness. This continuing for some days, he had taken pills, of a cathartic nature, every day for three days previous to his admission, and with the effect of producing an excessive action of the bowels, sometimes as many as a dozen discharges in twenty-four hours. The tongue was moderately coated, when examined by the class, countenance somewhat flushed, skin dry and husky, and pulse 90–100. The abdomen was rather tender on pressure, but the diarrhæa had been checked during the night by treatment. There was certainly nothing in the case to warrant the suspicion that a serious illness was impending; but the lecturer pointed out that the assemblage of symptoms marked a case of enteric fever, with inflammation of Peyer's glands and the solitary glands of the small intestines just on the verge of development. The inflammation was not yet manifest, but there was undoubtedly congestion,—an accumulation of blood in the glands, giving rise to irri tation, subsequent inflammation, softening and ulceration; the mesenteric glands, and, of course, the mucous membranes of the intestines being pretty uniformly affected, as well as the spleen, liver, and other large organs, in the progress of the disease. Cases of this kind, where purgation has been resorted to, need to be sedulously watched, for there is always a tenacious tendency to diarrhœa present in the typhoid fevers.

The treatment resorted to was the use of an emulsion of turpentine and laudanum in fʒj doses every four hours, to counteract and allay the morbid sensibility and exalted irritability along the course of the intestinal canal; and to incite an efficient action of the secretory system, and check the blood-degeneration, the fluid extract of *Asclepias Tuberosa* was given in fʒj doses, with chlorate of potash grs v, every four hours, alternating with the emulsion. Subsequently, this latter was changed to Dover's Powder grs v, hyd. sub mur. grj, with a view of producing an alterative influence on the glandular secretions, and with good effect. The mercurial was discontinued after the first twenty-four hours, and the emulsion kept steadily in use, together with a single powder of ipecac and opium grs v, every night at bed-time; the patient continuing to improve, and leaving his bed May 25th.

May 31st. The class were again assembled at his bed side, to find the patient well, to all appearance. But the lecturer stated that during his visit, two or three days previous, D. had complained of soreness and stiffness in one hip, so much so as to prevent his walking about. The seat of the pain was in the gluteal region, just above the level of the trochanter, and about midway between the trochanter and the sacrum. At first, apprehended it was nothing more than a cellular abscess, such as frequently follow recovery from fevers. Ordered an infusion of aconite leaves and muriate of ammonia to be applied, as a discutient and anodyne, with the dissimilar object of dissipating the tumor, if not too far advanced, or of hastening its formation, if too much progressed to be dispersed. At subsequent visits, failed to notice any change, and on more careful examination found all the symptoms of a low grade of inflammation in the capsular ligament of the hip-joint: in other words, an incipient case of *coxalgia* or hip-joint disease, brought on, undoubtedly, by too severe and too prolonged exercise, while the system was still enfeebled and depressed. There was no pain while the patient lay quiet, but severe pain felt on pressure of the femur into the acetabulum, or by striking the knee sharply upwards, or by abduction of the limb. The case not having advanced for enough to warrant the use of the instrument, and the condition favoring, strict rest was enjoined, and the use of the lotion already mentioned, continued; blisters, issues and other counter-irritants being deemed unadvisable, on account of the already exhausted condition of the patient. A bland, nutritious diet was advised, but no further medication.

June 7th, discharged cured.

